# Should initial ICU admission become a standard of care for acute bacterial meningitis ?

**DOI:** 10.1186/s13613-024-01277-3

**Published:** 2024-04-01

**Authors:** Michael Thy, Claire Dupuis, Jean-François Timsit, Romain Sonneville

**Affiliations:** 1 Medical and Infectious Diseases ICU, Bichat Claude Bernard University Hospital, Université Paris Cité, AP-HP, Paris, France; 2 EA7323, Pharmacology and Drug Evaluation in Children and Pregnant Women, Université Paris Cité, Paris, France; 3 Department of Intensive Care Medicine, Gabriel-Montpied University Hospital, Clermont- Ferrand, France; 4 Decision SCiences in Infectious Diseases control and care, UMR 1137-IAME Team:INSERM Université Paris Cité, Paris, 75018 France

The decision to admit adult patients with bacterial meningitis to the intensive care unit (ICU) is usually based on severe illness at hospital presentation, including altered mental status, seizures, respiratory and/or cardiovascular complications. However, as some patients may deteriorate rapidly within the first hours after hospital presentation, prophylactic ICU admission could be discussed in stable patients at high risk of secondary deterioration. However, the profile of these patients is not known. Two recent studies published in “Annals of Intensive Care” provide important information on this topic [1, 2]. A summary and comparison of the patients directly admitted to the ICU from the two studies are shown in Table 1.

**Table 1:** Comparison of the two studies for bacterial meningitis initially or directly admitted to the ICU

Direct ICU admission for bacterial meningitis	Chekrouni et al. [1]	Thy et al. [2]
Design	Prospective cohort of community-acquired bacterial meningitis (MeninGene study)	Retrospective analysis of pneumococcal meningitis with sepsis (PMSI database)
Country	Netherlands	France
Date of inclusion	From 2006 to 2022	From 2011 to 2020
**Main characteristics**	*N* = 2709	*N* = 4052
Initial/direct ICU admission rate	1369 (51%)	2006 (50%)
Median age (IQR), years	61 (49–69)	60 (49–70)
Male sex	737/1369 (54%)	1141 (57%)
*S. pneumoniae*	1071/1369 (78%)	100%
**Associated infection**		
Endocarditis	26/1312 (2%)	139 (7%)
Pneumonia	141/1313 (11%)	712 (36%)
**Complications**		
Coma (GCS < 8)	431/1360 (32%)	1117 (56%)
Focal neurological deficits	362 of 1220 (30%)	373 (19%)
Seizures	230/1313 (18%)	231 (12%)
Cranial nerve palsy / Brainstem failure	97/1074 (9%)	356 (18%)
Vascular complications*	244 (18%)	150 (8%)
Hydrocephalus	72/1241 (6%)	27 (1%)
**Organ failure on admission**		
Cardiovascular failure on admission	220/1272 (17%)	1188 (59%)
Respiratory failure on admission	470/1300 (36%)	1437 (72%)
**Outcomes**		
In-hospital mortality	304 (22%)	533 (27%)
Unfavorable outcome (GOS 2–4 or discharge to readaptation)	339 (25%)	337 (17%)
Median ICU length of stay (IQR), days	3 (2–8)	7 (3–15)
Median hospital length of stay (IQR), days	15 (12–24)	21 (13–37)

ICU: intensive or intermediate care unit, PMSI: Programme de Médicalisation des Systèmes d’Information, GCS: Glasgow coma scale, GOS: Glasgow outcome scale

*Vascular complications included ischemic stroke, transient ischemic stroke and cerebral venous thrombosis

The first study is the MeninGene nationwide study, conducted from 2006 to 2022 in the Netherlands, which presents a comprehensive examination of adults with community-acquired bacterial meningitis who eventually required ICU admission, either directly or secondarily [1]. This study provides precise estimates of intensive care needs in this population, with 51% of patients requiring initial ICU admission. This study confirms that the prognosis of such patients remains poor with high unfavorable outcome (47%) and hospital mortality (22%) rates. Factors independently associated with ICU admission were age, male gender, immunocompromised state, and factors associated with pneumococcal etiology. These factors may help to identify high risk patients and assist clinicians with complex decisions of ICU admission. Of particular concern, were the subgroup of patients initially admitted to non-ICU wards but subsequently transferred to the ICU, constituting 15% of cases. Importantly, this subgroup faced even higher rates of unfavorable outcome (66%) and mortality (30%) than those of directly admitted patients.

The second study based is a retrospective analysis of the French medico-administrative database on 4052 cases of pneumococcal meningitis with sepsis criteria recruited from 2011 to 2020 who eventually received care in the ICU [2]. Among them, 50% were directly admitted to the ICU and the remaining 50% were secondarily admitted. After adjustment for confounders (including gender, cardiovascular comorbidities, chronic obstructive pulmonary disease, diabetes, chronic kidney disease, neurological, respiratory and hematological failures on admission, associated diagnosis of endocarditis), direct ICU admission was associated with lower hospital mortality rates when compared to delayed admission (adjusted odds ratio 0.67, 95%CI 0.56–0.80). Although the reason for secondary ICU admission in both studies remains speculative, it is likely that these patients experienced early deterioration because of complications of neurologic or systemic origin. Adding the recent data from EURECA study and a recent large retrospective study [3, 4], we suggest a management algorithm of intensive care unit (ICU) admission for suspected or confirmed acute bacterial meningitis on Fig. 1.

**Fig. 1:**
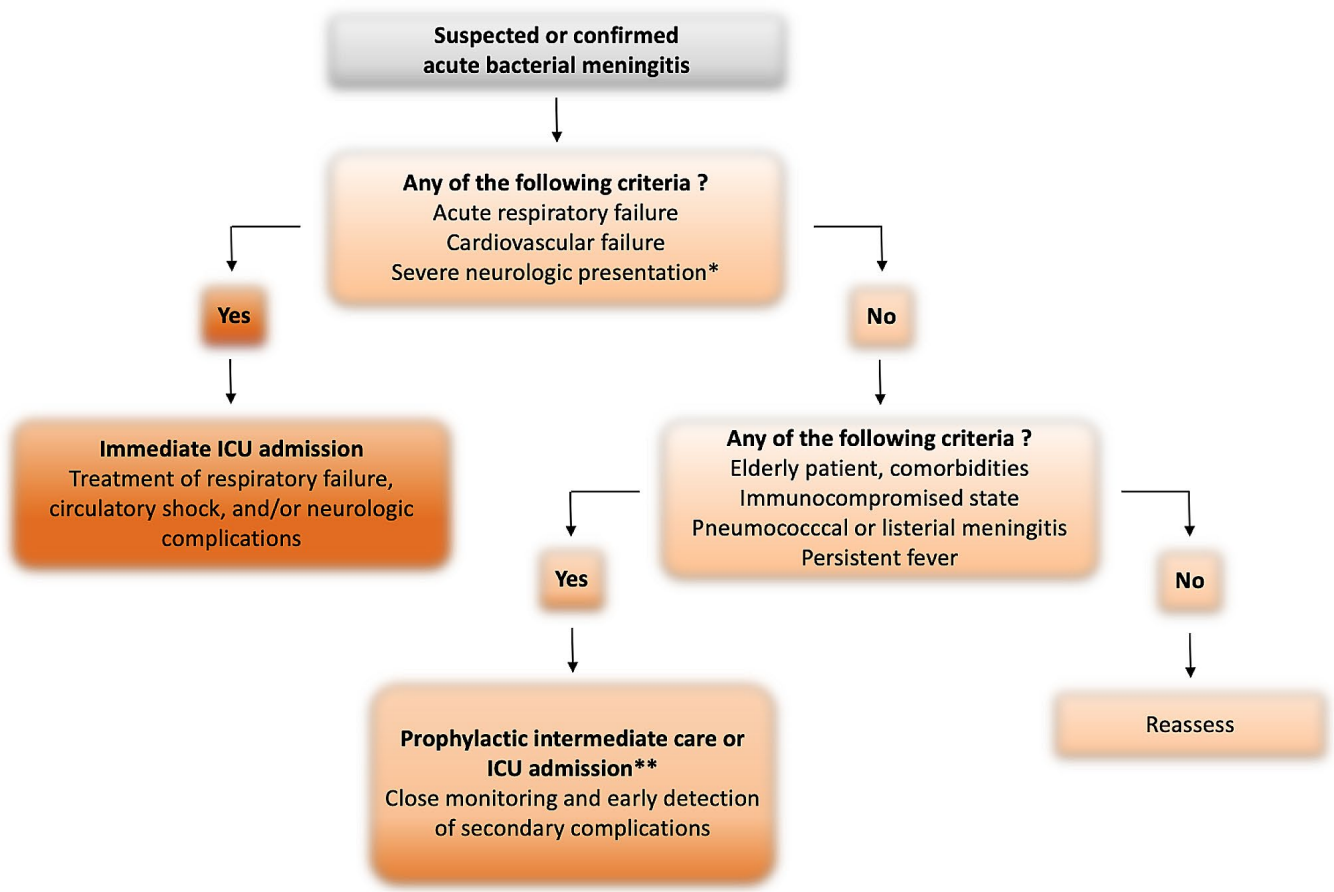
Management of intensive care unit (ICU) admission for suspected or confirmed acute bacterial meningitis. Legends: *Altered mental status, brainstem involvement/cranial nerve dysfunction and/or seizures. **With early discharge to medical wards after improvement

The implications of these two multicenter studies are profound as they help to identify patients at risk of poor outcome and to highlight the potential benefit of a more aggressive approach toward direct ICU admission, particularly for pneumococcal cases with sepsis. Indeed, such findings underscore the dynamic nature of patient trajectories and the urgency of refining admission protocols to ensure timely and appropriate care.

## Data Availability

Data available on request.
